# Bibliometric Analysis of Emerging Trends and Hotspots in the Links between Nonalcoholic Steatohepatitis and Diabetes Mellitus from 2004 to 2023

**DOI:** 10.2174/0118715303396313250904204350

**Published:** 2025-09-18

**Authors:** Chen Xie, Tao Liu, Yuanxin Zhong, Zhengyu Li, Ji Xu, Zijun Zhao, Xinqiang Wang, Po Gao

**Affiliations:** 1 Department of Anesthesiology, The First Affiliated Hospital of Huzhou University, Huzhou, Zhejiang, 313000, China;; 2 Department of Anesthesiology, Renji Hospital, Shanghai Jiao Tong University School of Medicine, Shanghai, 200127, China;; 3 Key Laboratory of Anesthesiology (Shanghai Jiao Tong University), Ministry of Education, Shanghai, China;; 4 Department of Anesthesiology, Chaohu Hospital Affiliated to Anhui Medical University, Chaohu, Anhui, China

**Keywords:** Nonalcoholic steatohepatitis, diabetes mellitus, bibliometric analysis, hotspots, citeSpace, antidiabetic drug therapy

## Abstract

**Introduction:**

In recent years, the prevalence of nonalcoholic steatohepatitis (NASH) has been rising globally. NASH has been linked to liver fibrosis, cirrhosis, hepatocellular carcinoma (HCC), and liver transplantation (LT), with the progression and severity of NASH closely impacting patients’ prognosis. This increasing incidence highlights the urgent need for effective therapeutic strategies and early detection methods to mitigate the progression of the disease and improve patient prognosis. Accumulating evidence indicates that NASH and diabetes mellitus (DM) are interconnected and mutually affect each other. This study utilized bibliometric analysis to assess current publication trends and focal points in the links between NASH and DM, aiming to promote research in this area.

**Methods:**

We thoroughly searched the Science Citation Index-Expanded (SCI-E) of the Web of Science Core Collection (WoSCC), PubMed, and the Excerpta Medica Database (Embase) to identify relevant articles on the links between NASH and DM from 2004 to 2023. The current publication trends and hotspots in this field were analyzed using the Online Analysis Platform of Literature Metrology, CiteSpace software, VOSviewer, and the R package Bibliometrix.

**Results:**

From 2004 to 2023, 943 articles were found that focused on the links between NASH and DM with a noticeable surge in publications since 2015. The United States has taken the primary position in terms of the number of publications. It has also been the most active country in international collaborative efforts. The University of California, San Diego, and Kenneth Cusi were the most productive institution and scholar, respectively. The co-citation keywords cluster labels revealed 10 primary clusters: adiponectin, MAFLD, mortality, NASH, nonalcoholic fatty liver, SGLT2, neurodegeneration, LY2405319, autophagy, and hepatocytes. Recent studies focused on weight loss, fibrosis stage, NAFLD, mortality, and diabetes mellitus.

**Discussion:**

Research on NASH and DM has transitioned from early mechanistic exploration to a current focus on weight management, diabetes control, and fibrosis prevention, particularly through lifestyle interventions and antidiabetic drug therapy. Future studies should integrate lifestyle adjustments with drug development, enhance international cooperation to fill regional research gaps, and achieve more effective management of NASH and DM.

**Conclusion:**

Over the past 20 years, global publications on the relationship between NASH and DM have grown rapidly. The current research hotspots focus on weight loss, and the reduction of blood glucose and fibrosis in NASH. Maintaining a healthy diet, exercising regularly, taking appropriate medication, and being vigilant about complications are essential for delaying the progression of NASH and DM. These are also the primary future directions of research.

**Registration Number:**

Registration number: 1020973(PROSPERO)

## 1 INTRODUCTION

Nonalcoholic steatohepatitis (NASH) is a dynamic condition that can regress to nonalcoholic fatty liver disease (NAFLD), remain constant, or progress to fibrosis and cirrhosis. The global prevalence of NASH is 3-6%, and 20% of cases progress from NAFLD [1, 2]. Various factors, such as lipotoxicity, insulin resistance (IR), and chronic inflammation, are involved in the development of NASH [3, 4]. Genetic determinants, comorbidities, and behavioral and environmental factors (such as exercise, diet, and socioeconomic status) can modulate these factors [5, 6]. Chronic low-grade inflammation, in which the spleen is the main contributor, is a central immune organ because many cells pass through the spleen rather than through all other secondary lymphoid organs combined [7]. Roughly 20% of individuals with NASH ultimately progress to cirrhosis [8, 9]. Furthermore, NASH is now becoming a major indication for liver transplantation (LT) worldwide [10, 11]. Moreover, the presence of clinically significant fibrosis in NASH is associated with cardiovascular events and mortality, and the annual all-cause mortality rate is 25.56‰ in NASH [12]. However, there are no licensed drugs available at present to prevent or treat the disease or to reverse its course. Progressing toward precision medicine for this intricate condition is proving to be quite challenging.

Diabetes mellitus (DM) is a significant global health concern, affecting individuals, healthcare services, and nations. In 2021, there were 537 million people aged 20 to 79 years worldwide living with DM. This number is projected to increase to 643 million by 2030 and 783 million by 2045 [13]. Over 90% of patients with diabetes mellitus have type 2 diabetes mellitus (T2DM). Persistent hyperglycemia in patients with DM leads to angiopathies, resulting in numerous progressive complications [14, 15]. These complications significantly increase the morbidity and mortality associated with diabetes worldwide [16, 17].

In patients with NASH, insulin cannot properly suppress glucose production, resulting in excessive glucose production [18]. Prolonged high levels of glucose can lead to liver dysfunction by triggering lipogenesis and fat accumulation [19, 20]. Consequently, individuals with T2DM exhibit a high incidence of liver steatosis and elevated serum levels of transaminases. The overall prevalence of T2DM in patients with NASH is 37.3% worldwide [21]. T2DM is the most common risk factor for the progression of NASH to liver fibrosis and hepatocellular carcinoma (HCC) [22]. Clinically, certain medications used for treating T2DM may be beneficial for the treatment of NASH. For instance, oral antidiabetic medications (*e.g.*, pioglitazone and empagliflozin) may hinder NASH progression in patients with T2DM [23-25]. Despite the fact that NASH is common among individuals with T2DM, it receives insufficient attention in clinical settings.

Bibliometric analysis is a quantitative tool that utilizes mathematical and statistical methods to provide a comprehensive overview of a specific research field [26-28]. This method has become more advanced and is now widely applied in various fields [29-33]. Nevertheless, despite reviewing a substantial amount of material, we found that no bibliometric studies have been conducted to evaluate the research trends and hotspots concerning the links between NASH and DM to date. The present study analyzed trends in publications and hotspots regarding the associations between NASH and DM from 2004 to 2023, offering a comprehensive overview for future studies.

## 2 MATERIALS AND METHODS

### 2.1 Database and Search Strategy

On May 17^th^, 2024, an extensive review of the literature was conducted using the Science Citation Index-Expanded (SCI-E) of the Web of Science Core Collection (WoSCC) database, PubMed, and the Excerpta Medica Database (Embase). Data from these databases contain comprehensive information for bibliometric analysis, including titles, authors, institutions, and references. However, sampling bias, citation delay, self-citation and journal indexing bias cannot be avoided for the period from 2004 to 2023. Meanwhile, to minimize bias from frequent database updates, data search and retrieval were both performed on the same day. The bibliometric analysis has been retrospectively registered at PROSPERO with registration number 1020973.

The inclusion criteria for retrieving relevant publications involved the following terms: (i) TS= ((“nonalcoholic steatohepatitis” OR “NASH” OR “non-alcoholic steatohepatitis” OR “non-alcoholic steatohepatitis”) AND (“diabetes” OR “diabetes mellitus” OR “mellitus” OR “diabetes mellitus, type 1” OR “type 1 diabetes” OR “T1DM” OR “diabetes mellitus, type 2” OR “type 2 diabetes” OR “T2DM”)); (ii) Publication Date= January 1^st^, 2004 to December 31st, 2023; (iii) Document type= Article; and (iv) Language= English. The chosen keywords were carefully selected to encompass a broad spectrum of research related to NASH and its association with various forms of diabetes, ensuring that both common and clinical terminologies were included [34-36]. Exclusion criteria were as follows: (i) no statements related to NASH or DM; (ii) document types (such as reviews, meta-analyses, letters, book chapters, editorial material, conference summaries, comments, *etc*.) other than scientific articles; and (iii) repeated publications. The selection process involved a rigorous screening of titles and abstracts to ensure that only studies meeting the established criteria were considered for full-text review. Each article's relevance was evaluated based on its contribution to understanding the relationship between NASH and DM, with particular attention given to the methodologies employed and the robustness of the findings. This systematic approach not only enhanced the reliability of the bibliometric analysis but also provided a structured framework for synthesizing the evidence. Raw data were obtained in the form of text files containing complete records. To validate the reliability of the results of the bibliometric analysis, Chen Xie and Ji Xu carefully assessed all publications based on publication years, titles, and abstracts. In case of disagreement, a third researcher, Po Gao, with 10 years of NAFLD/NASH research experience, reviewed the uncertain publication and made the final determination. The detailed filtering process is depicted in Fig. (**1**).

### 2.2 Bibliometric Analysis

After converting data to TXT format, they were imported into CiteSpace V6.2.R7 (Drexel University, Philadelphia, USA). We used the Online Analysis Platform of Literature Metrology (https://bibliometric.com/app), R package Bibliometrix for further analysis, and VOSviewer 1.6.19 (Leiden University, Leiden, NED). This was done to outline all the literature characteristics concerning the relationship between NASH and DM.

The Online Analysis Platform of Literature Metrology was used to extract the top 10 most productive journals and countries. Utilizing the R package Bibliometrix, a word cloud visualization was produced to display the top 100 high-frequency keywords. Furthermore, the interactions between countries were investigated using VOSviewer software. CiteSpace was utilized to generate several figures to better understand the relationship between NASH and DM and identify potential hotspots in this field. This included conducting co-citation analysis, assessing institutional collaboration and author collaboration, identifying citation bursts, analyzing keywords with the most significant citation bursts, and visualizing clustered networks of co-cited references. The visualization knowledge maps were formed using different sizes of nodes and links. Each map includes different nodes that symbolize entities like an organization, nation, referenced work, and author. Connections between nodes demonstrate the associations of co-citations or partnerships.

## 3 RESULTS

### 3.1 Quantity and Trends Analysis of Publications

**Fig. (1):**
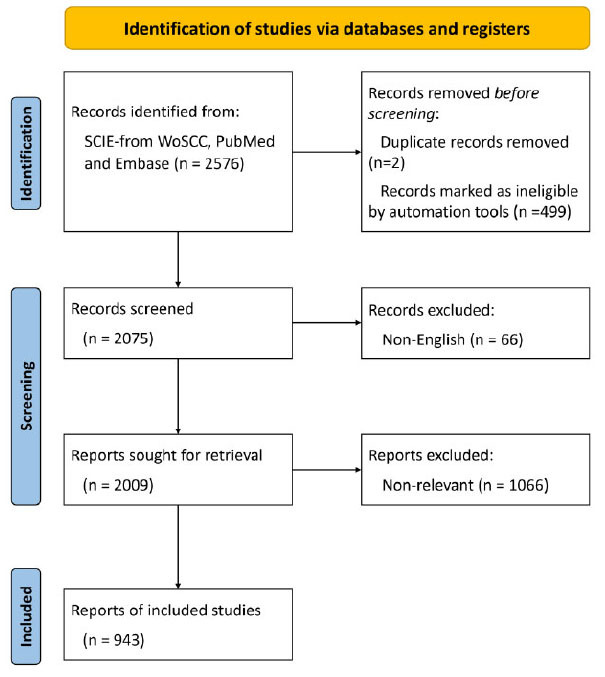
Flowchart outlining the inclusion or exclusion of publications.

**Fig. (2):**
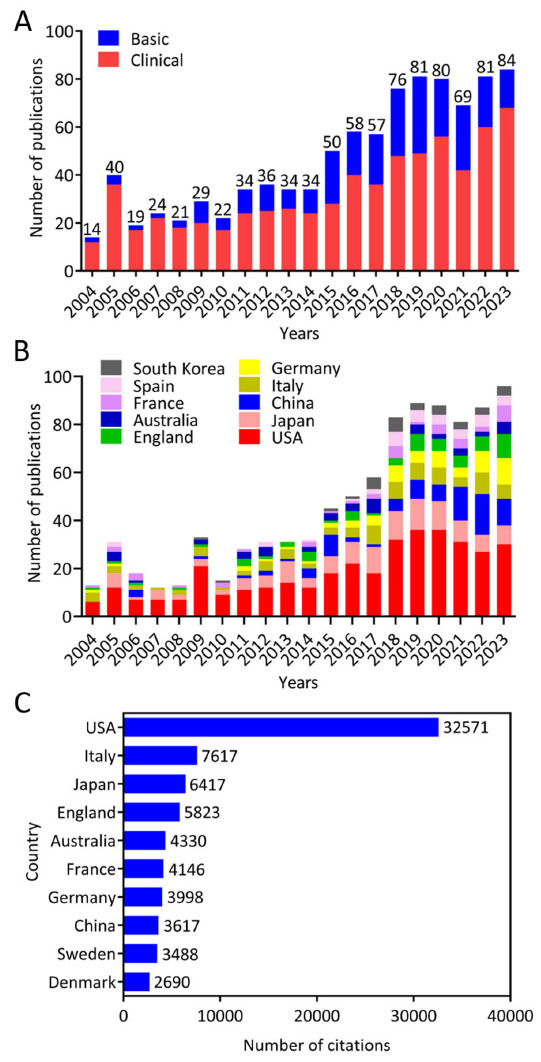
Reviewing the number and trends of published papers. (**A**) We extracted the annual number and type of publications on the link between NASH and DM from 2004 to 2023 based on data from the Web of Science. (**B**) Number of annual publications and growth trends in the link between NASH and DM from 2004 to 2023 based on data from the Online Analysis Platform of Literature Metrology. (**C**) The top 10 countries with the most citations.

In total, 2009 articles published in the SCI-E from 2004 to 2023 were included based on our criteria. After screening, 1066 publications were excluded due to irrelevance to our topic (Fig. **1**). The annual number of publications is displayed at the top of each bar in (Fig. **2A**), with different colors representing clinical studies or basic studies. From 2004 to 2014, there was a moderate increase in overall publication trend, although the number of publications in the next year was occasionally lower than that of the previous year. From 2015 to 2023, there has been a notable rise in publications, nearly three times the previous amount. Despite the decrease in the number of publications in 2021 compared to 2018-2020, the number of publications was still higher than in the first period. Moreover, Microsoft Excel 2021 was used to develop a growth trend model using the equation f(*x*)=0.1447*x*^2^+0.7162*x*^2^+18.861 (R^2^=0.8838), which predicted the publication of approximately 144 articles by 2030. Among all articles, 668 reported clinical research and 275 reported basic research. The annual number of clinical articles exceeded that of basic articles, and both types of research showed a similar growth trend in overall publication growth. Fig. (**2B**) displays the publication count from the top 10 countries over the last two decades. The United States dominated the field of NASH and DM with 368 publications, followed by Japan and China with a total number of 129 and 86 publications, respectively. The United States consistently led in terms of annual publications, with Japan maintaining a relatively stable number of publications each year. In contrast, China has experienced a rapid increase in terms of annual publications in the last five years.

As shown in Fig. (**2C**), the top 10 countries with the highest citation counts were determined by compiling the total number of citations for each country's publications. With 32571 citations, the United States occupied the top position in terms of the number of citations for articles published over a 20-year period. Italy and Japan occupied the second and third places with 7617 and 6417 citations, respectively. Table **1** ranks the top 10 countries with the highest mean number of citations for research on the links between NASH and DM from 2004 to 2023. Iceland, despite having only one article published, had the highest mean number of citations (1942). Norway and Scotland ranked second and third with 359.5 and 342.71 average citations, respectively. The average number of citations for the top 10 countries was at least 100 times higher. Although the United States, Japan, and China were among the top three countries in terms of total citations, their citations per article were 93.1, 50.12, and 36.86, respectively.

### 3.2 Analysis of Inter-Country and Inter-Institution Cooperation

**Fig. (3):**
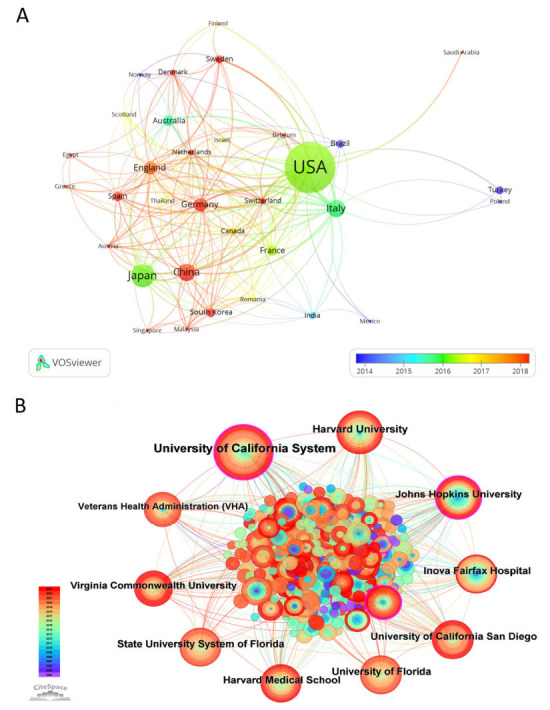
The collaboration among countries and institutions for investigating the link between NASH and DM from 2004 to 2023. (**A**) From 2004 to 2023, 64 countries collaborated to study the relationship between NASH and DM. (**B**) The institutions involved in research on the association between NASH and DM, emphasizing the top 10 most productive institutions.

To clarify the cooperation of countries or institutions in this area over the last two decades, we used VOSviewer software and CiteSpace for analysis. Between 2004 and 2023, a minimum of 1579 institutions across 64 countries/regions contributed to the publication of 943 articles. The results indicated that the United States was the most actively engaged country in international collaboration, followed by England, Germany, Italy, China, and Japan, respectively (Fig. **3A**).

The 10 most productive institutions are mapped out visually in Fig. (**3B**). The University of California, San Diego ranked first with the highest number of published papers (55), followed by Harvard Medical School (29), Johns Hopkins University (28), Inova Fairfax Hospital (26), University of California, San Fransisco (26), Harvard Medical School (25), State University System of Florida (25), University of Florida (25), Virginia Commonwealth University (22), and Veterans Health Administration (21). All of the 10 leading institutions were from the United States, indicating that American institutions played a pivotal role in research on the links between NASH and DM. The interconnected lines in Fig. (**3**) demonstrate the strong collaboration between different countries and institutions, highlighting the importance of collective efforts in advancing knowledge and treatment methods. The United States leads in output; other countries are gradually enhancing their contributions, fostering a more global approach to research in this field.

### 3.3 Core Author Distribution and Co-Authorship Networks

During the last two decades, at least 6519 authors made substantial contributions to the publication outputs, and Fig. (**4**) highlights the top 10 most productive authors. Kenneth Cusi from the University of Florida contributed the most articles (19), followed by Zobair M. Younossi from the Global NASH Council, Inova Healthy System (17), Fernando Bril from the University of Alabama Birmingham (14), Arun J. Sanyal from Virginia Commonwealth University (13), Rohit Loomba from the University of California, San Diego (12), Maria Stepanova from Johns Hopkins University (11), Naim Alkhouri from University of Arizona (10), Stephen A. Harrison from LSU AgCenter (9), Matthew M. Yeh from University of Washington (9), and Silvia Fargion from the University of Milan (8). These researchers have made outstanding contributions in the field of NASH and DM research. Kenneth Cusi, with the highest degree of centrality among researchers, is an endocrinologist who wields significant influence in the fields of diabetes, obesity, and metabolism [37]. He is a trailblazer in studying the connection between NAFLD/NASH and T2DM, highlighting the core role of IR in the pathogenesis of fatty liver and focuses on key therapeutic paths like “pioglitazone” and “prediabetes” [38]. His research orientation centers on metabolic abnormalities as the core target for NASH, leading to pioglitazone becoming the American Diabetes Association (ADA)-recommended treatment for NASH [39-41]. Zobair M. Younossi stands out in the author system for his high eigenvector centrality. He is a world-renowned hepatologist and public health expert, known for his groundbreaking work in NAFLD/NASH, liver disease epidemiology, health economics, and patient-reported outcomes (PROs) [42, 43]. He pioneered the global disease burden estimation model for NAFLD/NASH and created a research platform in hepatology and metabolic diseases [44-47]. The cooperative network map indicated that the most productive authors were closely connected through collaboration.

### 3.4 Analysis of Journals

Over the past two decades, 170 scholarly journals have published 943 original articles. Table **2** provides a list of the top 10 most-cited journals, including details on their citation frequency, impact factor (IF), and country of origin. The IF in the table only shows the most recent values due to limited space, so it is impossible to completely eliminate temporal bias or citation inflation. These journals primarily focused on hepatology, gastroenterology, and diabetes. Moreover, over half of these journals were from the United States. *Hepatology* ranked first with 7247 total citations, followed by *Journal of Hepatology* (6920) and *Gastroenterology* (5072). Four journals had an IF of more than 15, namely the *New England Journal of Medicine* (*NEJM*, 158.5), *Gastroenterology* (29.4), *Journal of Hepatology* (25.7), and *Diabetes Care* (16.2).

### 3.5 Analysis of Document Citations

The number of citations is a crucial factor in determining the influence of an article in a particular research field. We counted and ranked the citations for 943 articles, and the top 10 are displayed in Table **3**. The article with the highest number of citations was published in the *NEJM* in 2010 by Sanyal *et al*. from Virginia Commonwealth University [48]. This article has been cited 2244 times. This article demonstrated that vitamin E was superior to placebo in treating NASH in non-diabetic adults. It also indicated that pioglitazone may have potential efficacy. The second article, which was published in *Gastroenterology* and has received 1942 citations, supported the notion that NAFLD/NASH is independently associated with long-term overall mortality, LT, and liver-related outcomes [49]. With 1697 citations, the third article was published in *Hepatology* and indicated that most patients with NAFLD/NASH are likely to develop diabetes or impaired glucose tolerance. The study also indicated that survival is shorter in patients with NASH [50].

### 3.6 Analysis of Document Co-Citation and Clustered Network

The total number of references in 943 articles was 21994 (excluding self-citations) using CiteSpace. The visualization of co-cited references in Fig. (**5A**) showed the top 10 references. Table **4** highlights the top 10 references based on the frequency of citations they received. The highest-ranking cited reference was a narrative review published by *Hepatology* in 2018. This review presented a detailed analysis of the prevalence, pathogenesis, diagnosis, and management of NAFLD/NASH, based on the Practice Guidance from the American Association for the Study of Liver Diseases [51]. The references with the highest number of citations played a crucial role in demonstrating the link between NASH and DM and are widely recognized in this field.

By examining the emerging references in this field, we discovered research hotspots, as these references were frequently cited during a particular period. Fig. (**5B**) displays the top 25 references that experienced citation bursts in the field of NASH and DM from 2004 to 2023. The blue line represents a relatively unpopular time period from 2004 to 2023, while the red line represents the duration of the citation bursts.

The most recent reference that emerged was a clinical controlled trial published by the NEJM in 2021. In this study, 320 patients with NASH received semaglutide or placebo, and the findings indicated that a notably greater number of patients experienced NASH resolution in the semaglutide group compared to the placebo group [52]. The meta-analysis published by *Hepatology* in 2016 had the highest strength among these references, reaching 24.19. This burst started in 2017 and lasted until 2021. The global burden of NAFLD and NASH, including their prevalence, incidence, progression, and outcomes, was assessed in this meta-analysis, emphasizing the growing clinical and economic challenges associated with NAFLD due to the ongoing global rise in obesity rates [53].

By using the log-likelihood ratio algorithm in CiteSpace software, the co-citation map shown in Fig. (**6A**) was organized into clusters using keywords extracted from the references of 943 articles. Using hierarchical cluster labels, the analysis of co-citation clusters identified the key terms in the links between NASH and DM research, including #0 adiponectin, #1 MAFLD, #2 mortality, #3 NASH, #4 nonalcoholic fatty liver (NAFL), #5 SGLT2 (sodium-dependent glucose transporters 2), #6 neurodegeneration, #7 LY2405319 (an analog of FGF21, which lowers blood glucose), #8 autophagy, and #9 hepatocytes. There was an inverse relationship between the number of cluster labels and the number of articles in each cluster. Cluster #0, for example, had the highest number of papers compared to all other co-cited references.

Fig. (**6B**) provides a timeline view clearly showing the changes in hotspots in the links between NASH and DM over the last twenty years. Nonalcoholic fatty liver (NAFL) represented the most-cited cluster through the past two decades. The years 1999 to 2007 saw a high prevalence of co-citation with the keyword adiponectin, while from 2008 to 2016, mortality was the prevalent keyword in co-citation. In earlier years, novel biomarkers, such as adiponectin or adiponectin receptors (treatment targets of metabolic syndrome), opened up possibilities for personalized treatment options [54, 55]. As the timeline progresses, it becomes clear that there is a growing emphasis on comprehending the intricate relationship between metabolic disorders and liver health, with neurodegeneration and autophagy also becoming important areas of focus [56, 57]. The latest hotspots were MAFLD and SGLT2, which appeared in 2014 and 2013, respectively. They have persisted to date. The development of SGLT2 inhibitors (utilized in cases of β-cell dysfunction or IR), reflects a shift towards more efficient approaches in managing conditions of DM [58, 59]. This sustained interest highlights the evolving understanding of the interplay between metabolic disorders and liver health, underscoring the importance of continued research in these areas [60, 61].

### 3.7 Analysis of Research Trends and Keyword Burst

**Fig. (7):**
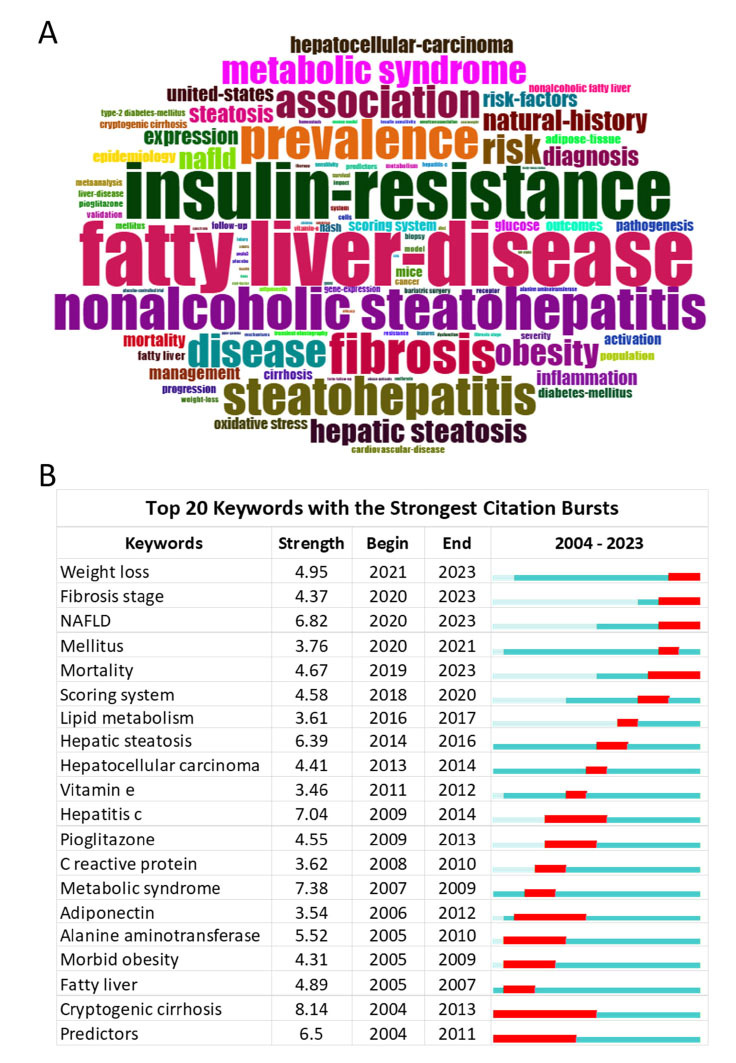
Examination of keywords and burst detection of publications regarding the links between NASH and DM from 2004 to 2023. (**A**) The R package bibliometrix was utilized to generate a word cloud displaying the top 100 most frequent keywords related to the links between NASH and DM. (**B**) Among the 943 citing articles on the links between NASH and DM, the keywords with the strongest burst strength are highlighted in red, signifying a sudden increase in usage frequency between 2004 and 2023. Keywords marked during that period are shown in blue to indicate a relatively less popular timeframe.

The top 100 frequently used keywords in studies on the links between NASH and DM are represented in the word cloud (Fig. **7A**). The size of the font increased with the frequency of each keyword. The keywords with high frequency included fatty liver disease, IR, NASH, fibrosis, and prevalence. Additionally, emerging terms such as metabolic syndrome, IR, and liver inflammation indicate a shift in focus towards understanding the complex interplay between these conditions. The analysis revealed notable bursts in certain keywords over the years, suggesting periods of heightened research activity and interest in specific aspects of the relationship between NASH and DM. This evolving landscape highlights the dynamic nature of the field and the ongoing efforts to uncover the underlying mechanisms and potential therapeutic targets. Keyword burst detection can help identify research hotspots. The top 20 keywords with the strongest bursts in the link between NASH and DM from 2004 to 2023 are shown in Fig. (**7B**). The blue line signifies a less favored timeframe between 2004 and 2023, while the red line shows the timeframe in which the burst keywords were sustained. The keyword burst, cryptogenic cirrhosis, lasted from 2004 to 2013 with the highest strength of 8.14. Since 2020, the latest keyword burst has focused on weight loss, fibrosis stage, NAFLD, mortality, and diabetes mellitus. The analysis points to a shift in research priorities, with a greater focus on weight management and halting disease progression in the context of metabolic disorders. The evolving landscape of keywords also reflects a growing recognition of the multifaceted nature of NASH and its association with diabetes, highlighting the importance of addressing both conditions in clinical practice and research [18, 62]. As these trends continue to develop, they may guide future investigations and therapeutic strategies aimed at mitigating the impact of these interconnected diseases on public health. Moreover, the keyword bursts may indicate important links to funding, regulation, or drug approval. The increase in keywords like hepatitis C, hepatic steatosis, diabetes mellitus, *etc*. may indicate a rise in research funding, such as grants from National Institutes of Health (NIH) [63, 64]. Simultaneously, the increase in mentions of adiponectin, pioglitazone, and vitamin E could lead regulatory agencies such as the Food and Drug Administration (FDA) to speed up the approval process for new drugs. These keywords' emergence may also indicate a rising interest in the scientific community, highlighting areas that are gaining attention and necessitating additional research, potentially influencing future research directions and funding priorities.

## 4 DISCUSSION

This study presents the first bibliometric analysis of the relationship between NASH and DM in the last two decades, providing a comprehensive review of the field. By using bibliometric analysis, researchers studying NASH and DM can gain a comprehensive overview of the field and easily identify the current research trends.

### 4.1 Overall Trends in Research on the Links Between NASH and DM

Over the past two decades, there has been a growing global interest in exploring the links between NASH and DM, with a noticeable surge in the number of publications since 2015. This increase in publications indicates a growing awareness of the clinical implications of the interplay between these two conditions, prompting researchers to delve deeper into their shared pathophysiological mechanisms, risk factors, and potential therapeutic targets. As the understanding of these conditions evolves, interdisciplinary collaborations are likely to develop, fostering innovative approaches to prevention and treatment. Furthermore, the rising incidence of NASH and DM in various populations highlights the urgent need for effective strategies to address their growing prevalence and associated healthcare burdens [65, 66]. The United States has played a leading role in NASH and DM research. Currently, the prevalence of NASH varies from 7% to 30%, affecting 28.9 million individuals in the United States and Europe [11]. NASH is emerging as the primary reason for LT among women and the second most common reason for LT among men in the United States [10, 11]. The high prevalence of both NASH and DM in the US has prompted researchers to focus on finding effective treatments and preventive measures. Additionally, the United States maintained its robust connections with other nations. The United States possesses the most productive institutions and the most influential experts, proving its pivotal role in global academic endeavors through both domestic and international cooperation.

It is important to note that research in this area is not limited to the US, as other countries such as Japan, China, and Germany have also made significant contributions. Due to the urgent need for disease prevention and treatment, and the significant advancements in the scientific research abilities of Chinese researchers, there has been a noticeable increase in the number of articles published in China over the past five years [67, 68]. However, the average number of publications on the link between NASH and DM in China (36.86) suggests that there is a need for improvement in the average quality of its research output. As the global prevalence of NASH and DM continues to increase, it is crucial for researchers from all countries to collaborate and share their knowledge to advance our understanding and treatment of these diseases.

The study conducted by Younossi *et al*. [69, 70] shows that South America and the Middle East and North Africa have the highest prevalence rates of NAFLD/NASH, with Africa having the lowest prevalence. Compared with countries with a high number of publications, some regions, such as Africa and South America have a lower number of publications. The relatively low output of publications in these regions can be attributed to weak infrastructure and brain-drain. Additionally, other diseases in these regions, such as HIV, have overshadowed NASH or DM in terms of attention [71, 72]. Addressing this gap is essential for developing inclusive research strategies that encompass a broader range of demographics and health disparities. On the other hand, the differences in the number of published papers among different countries and regions may be attributed to factors such as the allocation of special funds from institutions like the NIH. Collaborative efforts that prioritize inclusivity can facilitate the sharing of knowledge and resources, ultimately leading to more effective interventions tailored to diverse populations.

### 4.2 Transition of Research Hotspots on the Link between NASH and DM

From 2004 to 2014, studies mainly focused on the intricate mechanisms of NASH. Initially, it was discovered that adiponectin is involved in lipid metabolism, and reduced levels of adiponectin serve as an early warning sign for NASH. Hypoadiponectinemia can be identified before the development of diabetes and obesity and is associated with the severity of liver damage [73, 74]. Subsequently, cognitive impairment and neurodegeneration were discovered to complicate obesity, NASH, and T2DM, all of which are associated with IR [57]. The development of neurodegeneration in these diseases may be linked to the overproduction of neurotoxic ceramides in the liver, leading to IR in the brain [75]. Insulin-sensitizing agents, such as PPAR agonists, can be used as a therapeutic approach to prevent cognitive impairment [76]. Since 2010, an increasing number of studies have proposed several potential mechanistic roles for autophagy in NASH [77, 78]. Impaired autophagy can lead to fat accumulation in the liver and mitochondrial dysfunction, which in turn can worsen metabolic disturbances and accelerate the progression of NASH to end-stage liver disease [79, 80]. As our understanding of the mechanisms of NASH improves, the link between NASH and DM becomes more evident. Thus, it is essential to concomitantly treat NASH and DM. In the past five years, significant advances have been made in developing potential drugs for NASH. Takahashi *et al*. [81] indicated that ipragliflozin ameliorates obesity and hepatic outcomes in patients with DM and NAFLD/NASH, while larger studies are needed to confirm these effects. Pegozafermin, a glycosylated FGF21 analogue, showed significant promise as a therapeutic option for NASH and other metabolic disorders, warranting further studies [82, 83]. Given the complex etiology of NASH, experts have recommended the development of new treatments, such as RNA-based medications, to address genetic factors and inherited variants [84, 85]. However, the specific effects need to be further explored and verified. Hence, the use of hypoglycemic therapy to prevent NASH remains a prevailing trend for the foreseeable future.

The current keyword bursts focus on weight loss, fibrosis stage, NAFLD, mellitus, and mortality, suggesting that current studies primarily focus on the prevention and treatment of NASH *via* lifestyle interventions and modification of underlying metabolic conditions, such as diabetes. Researchers are also exploring the role of the microbiome in modulating liver health and its potential influence on NASH progression [86]. Additionally, ongoing clinical trials aim to assess the safety and efficacy of novel pharmacological agents alongside lifestyle modifications, emphasizing the importance of multidisciplinary care in managing these interconnected conditions [87, 88]. Ultimately, a comprehensive strategy that combines pharmacotherapy, dietary changes, and exercise may provide the most effective means of tackling NASH and its related metabolic complications [38, 89, 90]. A weight loss of more than 5% can ameliorate hepatic steatosis, while a weight loss of more than 10% can improve hepatic inflammation and fibrosis [91]. A recent clinical trial conducted by Stine **et al*.* in patients with NASH showed that Noom Weight, which includes self-monitoring of body weight, exercise, and food intake, is safe, feasible, and highly efficacious [92]. Noom Weight resulted in significantly greater weight loss compared to standard clinical care. The study by Koutoukidis *et al*. [90] revealed that weight change was independently and monotonically linked to changes in biochemical and histological features of NASH. As a result, guidelines for managing NASH should include suggestions for avoiding weight gain and providing support for weight loss. Although weight loss remains the first-line treatment for patients with NAFLD/NASH, the majority of individuals fail to sustain lifestyle changes. Sabench *et al*. [93] found that women with NASH who have diabetes tend to lose less weight after bariatric surgery compared to those without diabetes. This finding suggests that addressing underlying metabolic conditions may help in developing effective treatment strategies for NASH. More studies are needed to motivate patients to actively participate in their health management programs and to design individualized treatment strategies.

Over the past 20 years, fibrosis has become a hot topic. Substantial advancements have been made in exploring the mechanisms of fibrosis, with a focus on the molecular and cellular responses that lead to hepatocyte injury [94]. This damage triggers the release of cytokines, exacerbates inflammation and hepatocyte apoptosis, and leads to the accumulation of extracellular matrix, a characteristic of fibrosis [95]. Fibrosis increases the risk of NASH progression to cirrhosis, and fibrosis represents a major risk factor for HCC [96, 97]. Older patients with diabetes, particularly those with low serum albumin levels, should be monitored for the development of HCC [97]. Alexopoulos *et al*. [98] revealed that optimizing glycemic control can potentially modify the risk of NASH-related fibrosis . Ongoing studies have shown that alterations in the gut microbiome can contribute to the development of NASH, and targeting microbiome imbalances may hold promise for future treatments [99, 100]. With a deeper insight into the pathogenesis of fibrosis, it is believed that new targets or more effective treatments can be developed to improve patients’ outcomes [101]. Liver fibrosis will continue to be a hotspot in the field of NASH and DM in the coming years.

The growing body of research has not only highlighted the intricate pathways involved in fibrosis or IR but also emphasized the urgent need for translating these findings into clinical practice [102]. Despite the increasing number of studies, the gap between understanding the underlying mechanisms and developing new effective drugs remains significant. Given that there are no approved medications for fibrosis after NASH, it is imperative to develop effective treatments that can prevent or potentially reverse the progression of NASH and its complications. As researchers continue to investigate novel targets and treatment strategies, it is essential to align these efforts with clinical needs. Future research in the field of NASH and DM is likely to focus on developing innovative treatment strategies that combine lifestyle modifications, such as dietary adjustments and increased physical activity, with medications. Collaboration between healthcare providers, researchers, and pharmaceutical companies will be essential for translating these findings into clinical practice, with the ultimate goal of providing comprehensive care for individuals with NASH and DM and its associated complications.

### 4.3 Limitations

Our study had a few limitations. Firstly, we only used data from the SCI-E database of WoSCC, PubMed, and Embase, and did not use data from other important search engines. Data from these databases contain comprehensive information for bibliometric analysis, including titles, authors, institutions, and references. However, sampling bias, citation delay, self-citation, and journal indexing bias cannot be avoided. Secondly, bibliometric analysis is carried out using software to produce charts, which means that the limitations of the software are inevitable. An example of this is the issue of excessive clustering in CiteSpace. Thirdly, based on the literature inclusion and exclusion criteria, we excluded grey literature from our research articles. While it could potentially affect the overall results, it is unlikely to result in a substantial alteration. Fourthly, due to search parameters, study types, and language limitations, our search method might not cover all relevant articles. Furthermore, despite its frequent use in previous studies, relying on citations to assess the content and impact of an article has its own limitations. It is important to acknowledge and minimize these limitations in future studies.

## CONCLUSION

With the help of bibliometric mapping, we analyzed studies on the links between NASH and DM over the last two decades. The number of publications has steadily increased since 2015. The United States was the leader in this research field with the highest number of publications. It was actively involved in international cooperation, with high citation rates. The top 10 institutions were from the United States. Research on hypoglycemic drugs has become a major area for investigating the pathogenesis and treatment of NASH. The primary focus of current studies is on weight loss, reducing blood glucose, and preventing the progression of NASH to fibrosis. Future research in the field of NASH and DM is expected to concentrate on creating new treatment approaches that integrate lifestyle changes, like dietary modifications and enhanced physical activity, with medications. This bibliometric study delineated the general outlook for the links between NASH and DM, offering significant insights for current research endeavors and could help direct future NASH and DM research strategy.

## Figures and Tables

**Fig. (4):**
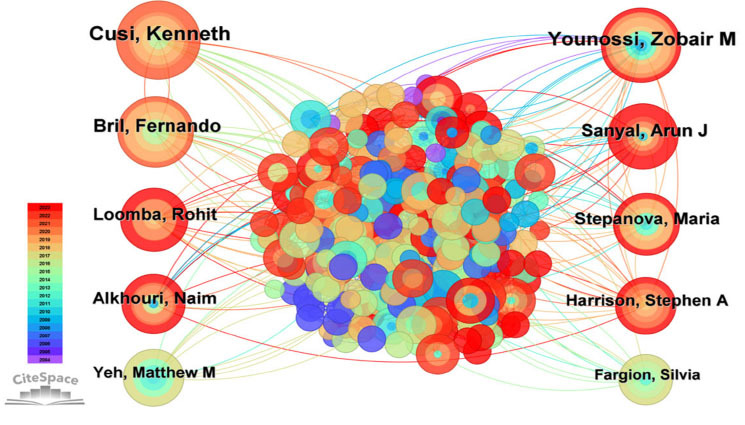
CiteSpace network displaying the authorship connections in the field of NASH and DM research, highlighting the top 10 authors with the most publications.

**Fig. (5):**
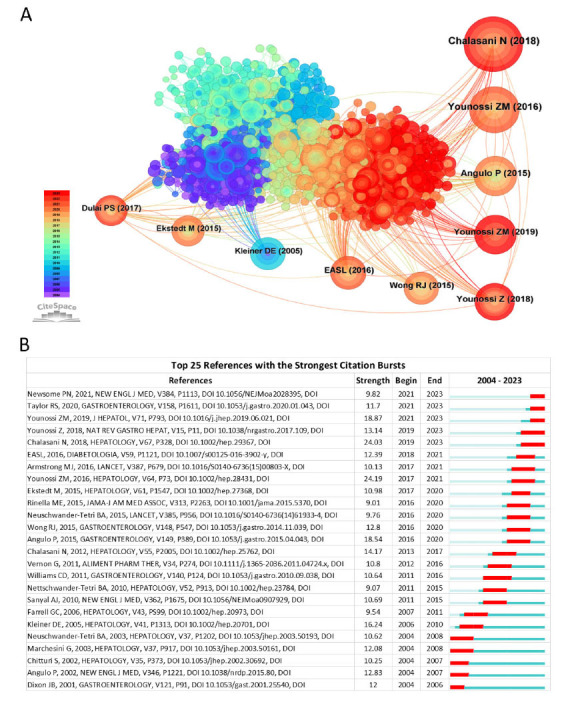
Reference co-citation network analysis of publications on the links between NASH and DM from 2004 to 2023. (**A**) The co-citation map created by CiteSpace includes 22,207 references pertaining to the links between NASH and DM. To only show the largest connected component, we conducted filtering, and 943 articles were retrieved for analysis. The top 10 references with the most cited publications are listed. (**B**) Among the 943 citing articles on the links between NASH and DM from 2004 to 2023, the reference with the strongest burst strength is denoted in red, signifying a sudden surge in usage frequency during that timeframe, and blue indicates a less popular period.

**Fig. (6):**
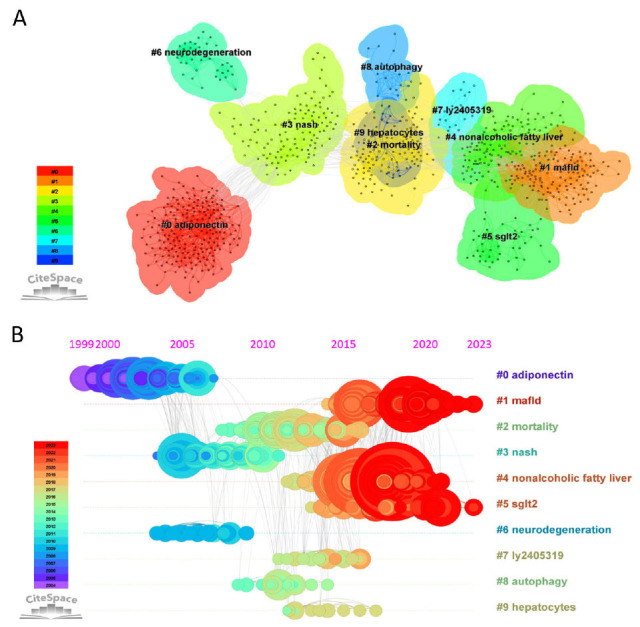
Reviewing the co-occurring keywords in the publications on the links between NASH and DM from 2004 to 2023. (**A**) The co-citation status of the reference under investigation and 943 citing articles was analyzed using CiteSpace to identify the top 10 largest clusters of co-cited references related to the links between NASH and DM. (**B**) The top 10 largest clusters of co-cited references on the links between NASH and DM are shown in a timeline view.

**Table 1:** The top 10 countries with the highest average number of citations per article from 943 retrieved articles related to the links between NASH and DM from 2004 to 2023.

Rank	Country	Number of Publications	Total Number of Citations	Average Number of Citations
1	Iceland	1	1942	1942.0
2	Norway	6	2157	359.5
3	Scotland	7	2399	342.7
4	Thailand	9	2190	243.3
5	Ukraine	1	150	150.0
8	Denmark	18	2690	149.4
7	Finland	5	674	134.8
6	Sweden	29	3652	125.9
9	Switzerland	22	2399	109.0
10	England	57	5823	102.2

**Note:** NASH, nonalcoholic steatohepatitis, DM, diabetes mellitus.

**Table 2:** The top 10 most active journals publishing articles on the links between NASH and DM from 2004 to 2023.

Rank	Journal Title	Frequency	Total Citations	Average Citation Per Paper	Average Citation per Year	Impact Factor (2024)	Country	JCR
1	Hepatology	40	7247	181.18	362.35	15.8	USA	Q1
2	Journal of Hepatology	34	6920	203.53	346	33.0	USA	Q1
3	Gastroenterology	11	5072	461.09	253.60	25.1	USA	Q1
4	New England Journal of Medicine	2	3566	1783	89.15	78.5	USA	Q1
5	Clinical Gastroenterology and Hepatology	11	2377	103.35	118.85	12.0	USA	Q1
6	American Journal of Gastroenterology	14	2032	145.14	101.6	7.6	USA	Q1
7	Diabetes Care	15	1619	107.93	80.95	16.6	USA	Q1
8	Liver International	29	1386	47.79	69.30	5.2	Denmark	Q1
9	Obesity Surgery	26	1172	45.08	58.60	3.1	Canada	Q2
10	Alimentary Pharmacology & Therapeutics	16	915	57.19	45.75	6.7	UK	Q2

**Note:** NASH, nonalcoholic steatohepatitis, DM, diabetes mellitus, JCR, Journal Citation Reports.

**Table 3:** The top 10 most cited articles from 943 retrieved articles on the links between NASH and DM from 2004 to 2023.

Rank	Title	First Author	Source	Year	Cited Frequency	DOI
1	Pioglitazone, Vitamin E, or Placebo for Nonalcoholic Steatohepatitis.	Sanyal AJ	*New England Journal of Medicine*	2010	2244	10.1056/NEJMoa0907929
2	Liver Fibrosis, but No Other Histologic Features, Is Associated With Long-term Outcomes of Patients With Nonalcoholic Fatty Liver Disease	Angulo P	*Gastroenterology*	2015	1942	10.1053/j.gastro.2015.04.043
3	Long-term follow-up of patients with NAFLD and elevated liver enzymes	Ekstedt M	*Hepatology*	2006	1697	10.1002/hep.21327
4	Prevalence of Nonalcoholic Fatty Liver Disease and Nonalcoholic Steatohepatitis Among a Largely Middle-Aged Population Utilizing Ultrasound and Liver Biopsy: A Prospective Study	Williams CD	*Gastroenterology*	2011	1578	10.1053/j.gastro.2010.09.038
5	A placebo-controlled trial of pioglitazone in subjects with nonalcoholic steatohepatitis	Belfort R	*New England Journal of Medicine*	2006	1322	10.1056/NEJMoa060326
6	Liraglutide safety and efficacy in patients with non-alcoholic steatohepatitis (LEAN): a multicentre, double-blind, randomised, placebo-controlled phase 2 study	Armstrong MJ	*Lancet*	2016	1225	10.1016/S0140-6736(15)00803-X
7	Modeling NAFLD disease burden in China, France, Germany, Italy, Japan, Spain, United Kingdom, and United States for the period 2016-2030	Estes C	*Journal of Hepatology*	2018	1046	10.1016/j.jhep.2018.05.036
8	NLRP3 inflammasome blockade reduces liver inflammation and fibrosis in experimental NASH in mice	Mridha AR	*Journal of Hepatology*	2017	683	10.1016/j.jhep.2017.01.022
9	Fibrosis stage but not NASH predicts mortality and time to development of severe liver disease in biopsy-proven NAFLD	Hagström H	*Journal of Hepatology*	2017	644	10.1016/j.jhep.2017.07.027
10	Long-Term Pioglitazone Treatment for Patients With Nonalcoholic Steatohepatitis and Prediabetes or Type 2 Diabetes Mellitus A Randomized Trial	Cusi K	*Annals of Internal Medicine*	2016	641	10.7326/M15-1774

**Table 4:** The top 10 most co-cited references of 943 retrieved articles related to the links between NASH and DM from 2004 to 2023.

Rank	Title	First Author	Journal	Year	Cited Frequency	DOI
1	The diagnosis and management of nonalcoholic fatty liver disease: Practice guidance from the American Association for the Study of Liver Diseases	Chalasani N	*Hepatology*	2018	89	10.1002/hep.29367
2	Global epidemiology of nonalcoholic fatty liver disease-Meta-analytic assessment of prevalence, incidence, and outcomes	Younossi ZM	*Hepatology*	2016	66	10.1002/hep.28431
3	Liver Fibrosis, but No Other Histologic Features, Is Associated With Long-term Outcomes of Patients With Nonalcoholic Fatty Liver Disease	Angulo P	*Gastroenterology*	2015	49	10.1053/j.gastro.2015.04.043
4	The global epidemiology of NAFLD and NASH in patients with type 2 diabetes: A systematic review and meta-analysis	Younossi ZM	*Journal of Hepatology*	2019	46	10.1016/j.jhep.2019.06.021
5	Global burden of NAFLD and NASH: trends, predictions, risk factors and prevention	Younossi Z	*Nature Reviews Gastroenterology & Hepatology*	2018	43	10.1038/nrgastro.2017.109
6	Nonalcoholic steatohepatitis is the second leading etiology of liver disease among adults awaiting liver transplantation in the United States	Wong RJ	*Gastroenterology*	2015	34	10.1053/j.gastro.2014.11.039
7	EASL-EASD-EASO Clinical Practice Guidelines for the management of non-alcoholic fatty liver disease	EASL-EASD-EASO	*Diabetologia*	2016	34	10.1007/S00125-016-3902-y
8	Design and validation of a histological scoring system for nonalcoholic fatty liver disease	Kleiner DE	*Hepatology*	2005	31	10.1002/hep.20701
9	Fibrosis stage is the strongest predictor for disease-specific mortality in NAFLD after up to 33 years of follow-up	Ekstedt M	*Hepatology*	2015	29	10.1002/hep.27368
10	Increased risk of mortality by fibrosis stage in nonalcoholic fatty liver disease: Systematic review and meta-analysis	Dulai PS	*Hepatology*	2017	29	10.1002/hep.29085

**Note:** The number of citation frequency means how many of the 943 articles cited a particular article as a reference. For instance, “89” means that 89 publications among the 943 articles cited “The diagnosis and management of nonalcoholic fatty liver disease: Practice guidance from the American Association for the Study of Liver Diseases” as one of their references.

## Data Availability

The data sets used and/or analysed during this study are available from the corresponding author upon request.

## LIST OF ABBREVIATIONS

NASH: Nonalcoholic Steatohepatitis
NAFLD: Nonalcoholic Fatty Liver Disease
HCC: Hepatocellular Carcinoma
IR: Insulin Resistance
LT: Liver Transplantation
DM: Diabetes Mellitus
T2DM: Type 2 Diabetes Mellitus
Woscc: Web of Science Core Collection
SCIE: Science Citation Index-Expanded
NEJM: New England Journal of Medicine
ADA: American Diabetes Association
NIH: National Institutes of Health
FDA: Food and Drug Administration

## STANDARDS OF REPORTING

PRISMA guidelines and methodology were followed.

## SUPPLEMENTARY MATERIAL

PRISMA checklist is available as supplementary material on the publisher’s website along with the published article.

## References

[1] Younossi Z.M., Golabi P., Paik J.M., Henry A., Van Dongen C., Henry L. (2023). The global epidemiology of nonalcoholic fatty liver disease (NAFLD) and nonalcoholic steatohepatitis (NASH): A systematic review.. Hepatology.

[2] Garg K., Brackett S., Hirsch I.B., Garg S.K. (2020). NAFLD/NASH and diabetes.. Diabetes Technol. Ther..

[3] Hosokawa Y., Hosooka T., Imamori M., Yamaguchi K., Itoh Y., Ogawa W. (2023). Adipose tissue insulin resistance exacerbates liver inflammation and fibrosis in a diet-induced NASH model.. Hepatol. Commun..

[4] Mendez-Sanchez N., Cruz-Ramon V.C., Ramirez-Perez O.L., Hwang J.P., Barranco-Fragoso B., Cordova-Gallardo J. (2018). New aspects of lipotoxicity in nonalcoholic steatohepatitis.. Int. J. Mol. Sci..

[5] Vuppalanchi R., Noureddin M., Alkhouri N., Sanyal A.J. (2021). Therapeutic pipeline in nonalcoholic steatohepatitis.. Nat. Rev. Gastroenterol. Hepatol..

[6] Yu S., Wang J., Zheng H., Wang R., Johnson N., Li T., Li P., Lin J., Li Y., Yan J., Zhang Y., Zhu Z., Ding X. (2022). Pathogenesis from inflammation to cancer in NASH-derived HCC.. J. Hepatocell. Carcinoma.

[7] Tarantino G., Citro V., Balsano C. (2021). Liver-spleen axis in nonalcoholic fatty liver disease.. Expert Rev. Gastroenterol. Hepatol..

[8] Sheka A.C., Adeyi O., Thompson J., Hameed B., Crawford P.A., Ikramuddin S. (2020). Nonalcoholic steatohepatitis.. JAMA.

[9] Canbay A., Sowa J.P., Syn W.K., Treckmann J. (2016). NASH Cirrhosis - the new burden in liver transplantation: how should it be managed?. Visc. Med..

[10] Garcia-Tsao G. (2021). Nonalcoholic Steatohepatitis — Opportunities and challenges.. N. Engl. J. Med..

[11] Cotter T.G., Charlton M. (2020). Nonalcoholic steatohepatitis after liver transplantation.. Liver Transpl..

[12] Llovet J.M., Willoughby C.E., Singal A.G., Greten T.F., Heikenwälder M., El-Serag H.B., Finn R.S., Friedman S.L. (2023). Nonalcoholic steatohepatitis-related hepatocellular carcinoma: pathogenesis and treatment.. Nat. Rev. Gastroenterol. Hepatol..

[13] Lim L.L., Chow E., Chan J.C.N. (2023). Cardiorenal diseases in type 2 diabetes mellitus: Clinical trials and real-world practice.. Nat. Rev. Endocrinol..

[14] Tilg H., Moschen A.R., Roden M. (2017). NAFLD and diabetes mellitus.. Nat. Rev. Gastroenterol. Hepatol..

[15] Liao H.Z., Liang Y., Wang Y., Liang C. (2023). Molecular pathology and therapeutic strategies of type 2 diabetes.. Endocr. Metab. Immune Disord. Drug Targets.

[16] GBD 2013 Mortality and Causes of Death Collaborators (2015). Global, regional, and national age–sex specific all-cause and cause-specific mortality for 240 causes of death, 1990–2013: a systematic analysis for the Global Burden of Disease Study 2013.. Lancet.

[17] Lean M.E.J. (2019). Low-calorie diets in the management of type 2 diabetes mellitus.. Nat. Rev. Endocrinol..

[18] Gastaldelli A., Cusi K. (2019). From NASH to diabetes and from diabetes to NASH: Mechanisms and treatment options.. JHEP Reports.

[19] Mantovani A., Byrne C.D., Bonora E., Targher G. (2018). Nonalcoholic fatty liver disease and risk of incident type 2 diabetes: a meta-analysis.. Diabetes Care.

[20] Petta S., Di Marco V., Pipitone R.M., Grimaudo S., Buscemi C., Craxì A., Buscemi S. (2018). Prevalence and severity of nonalcoholic fatty liver disease by transient elastography: Genetic and metabolic risk factors in a general population.. Liver Int..

[21] Younossi Z.M., Golabi P., de Avila L., Paik J.M., Srishord M., Fukui N., Qiu Y., Burns L., Afendy A., Nader F. (2019). The global epidemiology of NAFLD and NASH in patients with type 2 diabetes: A systematic review and meta-analysis.. J. Hepatol..

[22] Rinella M.E., Neuschwander-Tetri B.A., Siddiqui M.S., Abdelmalek M.F., Caldwell S., Barb D., Kleiner D.E., Loomba R. (2023). AASLD Practice Guidance on the clinical assessment and management of nonalcoholic fatty liver disease.. Hepatology.

[23] Cusi K., Orsak B., Bril F., Lomonaco R., Hecht J., Ortiz-Lopez C., Tio F., Hardies J., Darland C., Musi N., Webb A., Portillo-Sanchez P. (2016). Long-term Pioglitazone treatment for patients with nonalcoholic steatohepatitis and prediabetes or Type 2 Diabetes Mellitus.. Ann. Intern. Med..

[24] Lai L.L., Vethakkan S.R., Nik Mustapha N.R., Mahadeva S., Chan W.K. (2020). Empagliflozin for the treatment of nonalcoholic steatohepatitis in patients with type 2 diabetes mellitus.. Dig. Dis. Sci..

[25] Adeghate E.A. (2024). GLP-1 receptor agonists in the treatment of diabetic non–alcoholic steatohepatitis patients.. Expert Opin. Pharmacother..

[26] Li J., Wang L., Yin S., Yu S., Zhou Y., Lin X., Jiao Y., Yu W., Xia X., Yang L., Gao P. (2024). Emerging trends and hotspots of the itch research: A bibliometric and visualized analysis.. CNS Neurosci. Ther..

[27] Liao Y., Wang L., Liu F., Zhou Y., Lin X., Zhao Z., Xu S., Tang D., Jiao Y., Yang L., Yu W., Gao P. (2023). Emerging trends and hotspots in metabolic dysfunction-associated fatty liver disease (MAFLD) research from 2012 to 2021: A bibliometric analysis.. Front. Endocrinol. (Lausanne).

[28] Lin X., Zhou Y., Ye L., Wang B., Jiao Y., Yu W., Gao P., Yang L. (2023). A bibliometric and visualized analysis of hepatic ischemia-reperfusion injury (HIRI) from 2002 to 2021.. Heliyon.

[29] Xie C., Piao M., Zhou L., Tao X., Yao Y., Jiang B., Wang X., Yan M. (2025). Emerging trends and hotspots in animal experimental research on lung transplantation from 2004 to 2023: A bibliometric analysis.. J. Thorac. Dis..

[30] Li D., Liu Y., Hui Y., Li B., Hao C. (2025). A glimpse of research trends and frontiers in the etiology of premature ovarian insufficiency *via* bibliometric analysis.. Endocr. Metab. Immune Disord. Drug Targets.

[31] Wu Y., Cai T., Tao Y., Zhao J., Zhang J. (2024). Emerging insights and global trends in the relationship between selenium and thyroid diseases: A bibliometric analysis.. Endocr. Metab. Immune Disord. Drug Targets.

[32] Mao M., Zhou Y., Jiao Y., Yin S., Cheung C., Yu W., Gao P., Yang L. (2022). Bibliometric and visual analysis of research on the links between the gut microbiota and pain from 2002 to 2021.. Front. Med. (Lausanne).

[33] Cao X., Li F., Xie X., Ling G., Tang X., He W., Tian J., Ge Y. (2025). Efferocytosis and inflammation: A bibliometric and systematic analysis.. Front. Med. (Lausanne).

[34] Li X., Su X., Xia F., Qiu J., Zhang J., Wu H., Xie X., Xu M. (2023). Bibliometric and visual analysis of diabetes mellitus and pyroptosis from 2011 to 2022.. Eur. J. Med. Res..

[35] Kong L., Deng B., Guo M., Chen M., Wang X., Zhang M., Tang H., Wang Q., Yang L., Xiong Z. (2023). A systematic bibliometric analysis on the clinical practice of CGM in diabetes mellitus from 2012 to 2022.. Front. Endocrinol. (Lausanne).

[36] Pezzino S., Sofia M., Mazzone C., Castorina S., Puleo S., Barchitta M., Agodi A., Gallo L., La Greca G., Latteri S. (2023). Gut Microbiome in the progression of nafld, nash and cirrhosis, and its connection with biotics: A bibliometric study using dimensions scientific research database.. Biology.

[37] Cusi K. (2020). Time to include nonalcoholic steatohepatitis in the management of patients with type 2 diabetes.. Diabetes Care.

[38] Cusi K. (2020). A diabetologist’s perspective of non-alcoholic steatohepatitis (NASH): Knowledge gaps and future directions.. Liver Int..

[39] Cusi K. (2018). Pioglitazone for the treatment of NASH in patients with prediabetes or type 2 diabetes mellitus.. Gut.

[40] Cusi K. (2012). Role of obesity and lipotoxicity in the development of nonalcoholic steatohepatitis: Pathophysiology and clinical implications.. Gastroenterology.

[41] Cusi K. (2024). Nonalcoholic fatty liver disease in diabetes: A call to action.. Diabetes Spectr..

[42] Younossi Z.M., Stepanova M., Lawitz E., Charlton M., Loomba R., Myers R.P., Subramanian M., McHutchison J.G., Goodman Z. (2018). Improvement of hepatic fibrosis and patient-reported outcomes in non-alcoholic steatohepatitis treated with selonsertib.. Liver Int..

[43] Younossi Z.M., Stepanova M., Noureddin M., Kowdley K.V., Strasser S.I., Kohli A., Ruane P., Shiffman M.L., Sheikh A., Gunn N., Caldwell S.H., Huss R.S., Myers R.P. (2021). Improvements of fibrosis and disease activity are associated with improvement of patient-reported outcomes in patients with advanced fibrosis due to nonalcoholic steatohepatitis.. Hepatol. Commun..

[44] Mishra A., Younossi Z.M. (2012). Epidemiology and natural history of non-alcoholic fatty liver disease.. J. Clin. Exp. Hepatol..

[45] Younossi Z.M. (2017). Long-term outcomes of nonalcoholic fatty liver disease: From nonalcoholic steatohepatitis to nonalcoholic steatofibrosis.. Clin. Gastroenterol. Hepatol..

[46] Younossi Z.M. (2024). Predicting liver-related outcomes in steatotic liver disease.. JAMA.

[47] Younossi Z.M. (2019). Non-alcoholic fatty liver disease: A global public health perspective.. J. Hepatol..

[48] Sanyal A.J., Chalasani N., Kowdley K.V., McCullough A., Diehl A.M., Bass N.M., Neuschwander-Tetri B.A., Lavine J.E., Tonascia J., Unalp A., Van Natta M., Clark J., Brunt E.M., Kleiner D.E., Hoofnagle J.H., Robuck P.R., NASH CRN (2010). Pioglitazone, vitamin E, or placebo for nonalcoholic steatohepatitis.. N. Engl. J. Med..

[49] Angulo P., Kleiner D.E., Dam-Larsen S., Adams L.A., Bjornsson E.S., Charatcharoenwitthaya P., Mills P.R., Keach J.C., Lafferty H.D., Stahler A., Haflidadottir S., Bendtsen F. (2015). Liver Fibrosis, but no other histologic features, is associated with long-term outcomes of patients with nonalcoholic fatty liver disease.. Gastroenterology.

[50] Ekstedt M., Franzén L.E., Mathiesen U.L., Thorelius L., Holmqvist M., Bodemar G., Kechagias S. (2006). Long-term follow-up of patients with NAFLD and elevated liver enzymes†.. Hepatology.

[51] Chalasani N., Younossi Z., Lavine J.E., Charlton M., Cusi K., Rinella M., Harrison S.A., Brunt E.M., Sanyal A.J. (2018). The diagnosis and management of nonalcoholic fatty liver disease: Practice guidance from the American association for the study of liver diseases.. Hepatology.

[52] Newsome P.N., Buchholtz K., Cusi K., Linder M., Okanoue T., Ratziu V., Sanyal A.J., Sejling A.S., Harrison S.A., NN9931-4296 Investigators (2021). A placebo-controlled trial of subcutaneous semaglutide in nonalcoholic steatohepatitis.. N. Engl. J. Med..

[53] Younossi Z.M., Koenig A.B., Abdelatif D., Fazel Y., Henry L., Wymer M. (2016). Global epidemiology of nonalcoholic fatty liver disease-Meta-analytic assessment of prevalence, incidence, and outcomes.. Hepatology.

[54] Kadowaki T., Yamauchi T. (2005). Adiponectin and adiponectin receptors.. Endocr. Rev..

[55] Kadowaki T., Yamauchi T., Kubota N., Hara K., Ueki K. (2007). Adiponectin and adiponectin receptors in obesity-linked insulin resistance.. Novartis Found. Symp..

[56] Das S., Seth R.K., Kumar A., Kadiiska M.B., Michelotti G., Diehl A.M., Chatterjee S. (2013). Purinergic receptor X7 is a key modulator of metabolic oxidative stress-mediated autophagy and inflammation in experimental nonalcoholic steatohepatitis.. Am. J. Physiol. Gastrointest. Liver Physiol..

[57] Monte S.M., Longato L. (2009). Nitrosamine exposure exacerbates high fat diet-mediated type 2 diabetes mellitus, non-alcoholic steatohepatitis, and neurodegeneration with cognitive impairment.. Mol. Neurodegener..

[58] Dardi I., Kouvatsos T., Jabbour S.A. (2016). SGLT2 inhibitors.. Biochem. Pharmacol..

[59] O’Hara D.V., Jardine M.J. (2022). SGLT2 inhibitors may prevent diabetes.. Nat. Rev. Nephrol..

[60] Mentsiou N. (2024). The Interplay between endocrine-disrupting chemicals and the epigenome towards metabolic dysfunction-associated steatotic liver disease: A comprehensive review.. Nutrients.

[61] Zhang Z., Qin X., Yi T., Li Y., Li C., Zeng M., Luo H., Lin X., Xie J., Xia B., Lin Y., Lin L. (2024). Gubra Amylin-NASH diet induced nonalcoholic fatty liver disease associated with histological damage, oxidative stress, immune disorders, gut microbiota, and its metabolic dysbiosis in colon.. Mol. Nutr. Food Res..

[62] Chianelli M., Armellini M., Carpentieri M., Coccaro C., Cuttica C.M., Fusco A., Marucci S., Nelva A., Nizzoli M., Ponziani M.C., Sciaraffia M., Tassone F., Busetto L. (2025). Obesity in prediabetic patients: Management of metabolic complications and strategies for prevention of overt diabetes.. Endocr. Metab. Immune Disord. Drug Targets.

[63] Bahirwani R., Barin B., Olthoff K., Stock P., Murphy B., Rajender Reddy K., Solid Organ Transplantation in HIV: Multi-Site Study Investigators (2013). Chronic kidney disease after liver transplantation in human immunodeficiency virus/hepatitis C virus–coinfected recipients *versus* human immunodeficiency virus–infected recipients without hepatitis C virus: Results from the national institutes of health multi-site study.. Liver Transpl..

[64] Perry A.S., Annis J.S., Master H., Nayor M., Hughes A., Kouame A., Natarajan K., Marginean K., Murthy V., Roden D.M., Harris P.A., Shah R., Brittain E.L. (2023). Association of longitudinal activity measures and diabetes risk: an analysis from the national institutes of health *All of Us* Research Program.. J. Clin. Endocrinol. Metab..

[65] Younossi Z.M., Tampi R.P., Racila A., Qiu Y., Burns L., Younossi I., Nader F. (2020). Economic and clinical burden of nonalcoholic steatohepatitis in patients with type 2 diabetes in the U.S.. Diabetes Care.

[66] Fishman J., Tapper E.B., Dodge S., Miller K., Lewandowski D., Bogdanov A., Bonafede M. (2023). The incremental cost of non-alcoholic steatohepatitis and type 2 diabetes in the United States using real-world data.. Curr. Med. Res. Opin..

[67] Zou H., Ge Y., Lei Q., Ung C.O.L., Ruan Z., Lai Y., Yao D., Hu H. (2022). Epidemiology and disease burden of non-alcoholic steatohepatitis in greater China: A systematic review.. Hepatol. Int..

[68] Lu R., Liu Y., Hong T. (2023). Epidemiological characteristics and management of nonalcoholic fatty liver disease/nonalcoholic steatohepatitis in China: A narrative review.. Diabetes Obes. Metab..

[69] Golabi P., Owrangi S., Younossi Z.M. (2024). Global perspective on nonalcoholic fatty liver disease and nonalcoholic steatohepatitis - prevalence, clinical impact, economic implications and management strategies.. Aliment. Pharmacol. Ther..

[70] Younossi Z.M., Golabi P., Paik J., Owrangi S., Yilmaz Y., El-Kassas M., Alswat K., Alqahtani S.A. (2024). Prevalence of metabolic dysfunction-associated steatotic liver disease in the Middle East and North Africa.. Liver Int..

[71] Satoh S., Boyer E. (2019). HIV in South Africa.. Lancet.

[72] Cohen J. (2006). HIV/AIDS: Latin America & Caribbean. South America.. Science.

[73] Musso G., Gambino R., Durazzo M., Biroli G., Carello M., Fagà E., Pacini G., De Michieli F., Rabbione L., Premoli A., Cassader M., Pagano G. (2005). Adipokines in NASH: postprandial lipid metabolism as a link between adiponectin and liver disease.. Hepatology.

[74] Larter C.Z., Farrell G.C. (2006). Insulin resistance, adiponectin, cytokines in NASH: Which is the best target to treat?. J. Hepatol..

[75] Lyn-Cook L.E., Lawton M., Tong M., Silbermann E., Longato L., Jiao P., Mark P., Wands J.R., Xu H., de la Monte S.M. (2009). Hepatic ceramide may mediate brain insulin resistance and neurodegeneration in type 2 diabetes and non-alcoholic steatohepatitis.. J. Alzheimers Dis..

[76] de la Monte S.M., Longato L., Tong M., Wands J.R. (2009). Insulin resistance and neurodegeneration: Roles of obesity, type 2 diabetes mellitus and non-alcoholic steatohepatitis.. Curr. Opin. Investig. Drugs.

[77] Masouminia M., Samadzadeh S., Mendoza A.S., French B.A., Tillman B., French S.W. (2016). Upregulation of autophagy components in alcoholic hepatitis and nonalcoholic steatohepatitis.. Exp. Mol. Pathol..

[78] Amir M., Czaja M.J. (2011). Autophagy in nonalcoholic steatohepatitis.. Expert Rev. Gastroenterol. Hepatol..

[79] Udoh U.A.S., Rajan P.K., Nakafuku Y., Finley R., Sanabria J.R. (2022). Cell autophagy in NASH and NASH-related hepatocellular carcinoma.. Int. J. Mol. Sci..

[80] Fucho R., Martínez L., Baulies A., Torres S., Tarrats N., Fernandez A., Ribas V., Astudillo A.M., Balsinde J., Garcia-Rovés P., Elena M., Bergheim I., Lotersztajn S., Trautwein C., Appelqvist H., Paton A.W., Paton J.C., Czaja M.J., Kaplowitz N., Fernandez-Checa J.C., García-Ruiz C. (2014). ASMase regulates autophagy and lysosomal membrane permeabilization and its inhibition prevents early stage non-alcoholic steatohepatitis.. J. Hepatol..

[81] Takahashi H., Kessoku T., Kawanaka M., Nonaka M., Hyogo H., Fujii H., Nakajima T., Imajo K., Tanaka K., Kubotsu Y., Isoda H., Oeda S., Kurai O., Yoneda M., Ono M., Kitajima Y., Tajiri R., Takamori A., Kawaguchi A., Aishima S., Kage M., Nakajima A., Eguchi Y., Anzai K. (2022). Ipragliflozin improves the hepatic outcomes of patients with diabetes with NAFLD.. Hepatol. Commun..

[82] Loomba R., Lawitz E.J., Frias J.P., Ortiz-Lasanta G., Johansson L., Franey B.B., Morrow L., Rosenstock M., Hartsfield C.L., Chen C.Y., Tseng L., Charlton R.W., Mansbach H., Margalit M. (2023). Safety, pharmacokinetics, and pharmacodynamics of pegozafermin in patients with non-alcoholic steatohepatitis: A randomised, double-blind, placebo-controlled, phase 1b/2a multiple-ascending-dose study.. Lancet Gastroenterol. Hepatol..

[83] Tseng C.M.L., Balic K., Charlton R.W., Margalit M., Mansbach H., Savic R.M. (2023). Population pharmacokinetics and pharmacodynamics of pegozafermin in patients with nonalcoholic steatohepatitis.. Clin. Pharmacol. Ther..

[84] Wei S., Wang L., Evans P.C., Xu S. (2024). NAFLD and NASH: Etiology, targets and emerging therapies.. Drug Discov. Today.

[85] Wen Y., Ju C. (2020). MER proto-oncogene tyrosine kinase: A novel potential target to treat nonalcoholic steatohepatitis fibrosis.. Hepatology.

[86] Kolodziejczyk A.A., Zheng D., Shibolet O., Elinav E. (2019). The role of the microbiome in NAFLD and NASH.. EMBO Mol. Med..

[87] Dewidar B., Kahl S., Pafili K., Roden M. (2020). Metabolic liver disease in diabetes – From mechanisms to clinical trials.. Metabolism.

[88] Lee K.W., Devaraj N.K., Ching S.M., Veettil S.K., Hoo F.K., Deuraseh I., Soo M.J. (2021). Effect of SGLT-2 inhibitors on non-alcoholic fatty liver disease among patients with type 2 diabetes mellitus: Systematic review with meta-analysis and trial sequential analysis of randomized clinical trials.. Oman Med. J..

[89] Panasevich M.R., Peppler W.T., Oerther D.B., Wright D.C., Rector R.S. (2017). Microbiome and NAFLD: potential influence of aerobic fitness and lifestyle modification.. Physiol. Genomics.

[90] Koutoukidis D.A., Jebb S.A., Aveyard P., Astbury N.M. (2020). The effect of moderate weight loss on a non-invasive biomarker of liver fibrosis: A randomised controlled trial.. Obes. Facts.

[91] Malespin M.H., Barritt A.S., Watkins S.E., Schoen C., Tincopa M.A., Corbin K.D., Mospan A.R., Munoz B., Trinh H.N., Weiss L.M., Reddy K.R., Loomba R., Kemmer N., Lok A.S. (2022). Weight loss and weight regain in usual clinical practice: results from the TARGET-NASH Observational Cohort.. Clin. Gastroenterol. Hepatol..

[92] Stine J.G., Rivas G., Hummer B., Duarte-Rojo A., May C.N., Geyer N., Chinchilli V.M., Conroy D.E., Mitchell E.S., McCallum M., Michealides A., Schmitz K.H. (2023). Mobile health lifestyle intervention program leads to clinically significant loss of body weight in patients with NASH.. Hepatol. Commun..

[93] Sabench F., Bertran L., Vives M., París M., Aguilar C., Martínez S., Binetti J., Real M., Alibalic A., Richart C., del Castillo D., Auguet T. (2022). Nash presence is associated with a lower weight loss one and 2 years after bariatric surgery in women with severe obesity.. Obes. Surg..

[94] Parola M., Pinzani M. (2024). Liver fibrosis in NAFLD/NASH: From pathophysiology towards diagnostic and therapeutic strategies.. Mol. Aspects Med..

[95] Roehlen N., Crouchet E., Baumert T.F. (2020). Liver fibrosis: Mechanistic concepts and therapeutic perspectives.. Cells.

[96] Farrell G.C., Larter C.Z. (2006). Nonalcoholic fatty liver disease: From steatosis to cirrhosis.. Hepatology.

[97] Yang J.D., Ahmed F., Mara K.C., Addissie B.D., Allen A.M., Gores G.J., Roberts L.R. (2020). Diabetes Is associated with increased risk of hepatocellular carcinoma in patients with cirrhosis from nonalcoholic fatty liver disease.. Hepatology.

[98] Alexopoulos A.S., Crowley M.J., Wang Y., Moylan C.A., Guy C.D., Henao R., Piercy D.L., Seymour K.A., Sudan R., Portenier D.D., Diehl A.M., Coviello A.D., Abdelmalek M.F. (2021). Glycemic control predicts severity of hepatocyte ballooning and hepatic fibrosis in nonalcoholic fatty liver disease.. Hepatology.

[99] Milosevic I., Vujovic A., Barac A., Djelic M., Korac M., Radovanovic Spurnic A., Gmizic I., Stevanovic O., Djordjevic V., Lekic N., Russo E., Amedei A. (2019). Gut-Liver Axis, gut microbiota, and its modulation in the management of liver diseases: A review of the literature.. Int. J. Mol. Sci..

[100] Liu Q., Liu S., Chen L., Zhao Z., Du S., Dong Q., Xin Y., Xuan S. (2019). Role and effective therapeutic target of gut microbiota in NAFLD/NASH (Review).. Exp. Ther. Med..

[101] Huisman T.M., Dieterich D.T., Friedman S.L. (2021). Experimental and investigational targeted therapies for the management of fibrosis in nash: An update.. J. Exp. Pharmacol..

[102] Lin S.Z., Fan J.G. (2022). Peripheral immune cells in NAFLD patients: A spyhole to disease progression.. EBioMedicine.

